# Ethnic Identity and Genome Wide Runs of Homozygosity

**DOI:** 10.1007/s10519-021-10053-z

**Published:** 2021-03-16

**Authors:** Martin Fieder, Brittany L. Mitchell, Scott Gordon, Susanne Huber, Nicholas G. Martin

**Affiliations:** 1 Department of Evolutionary Anthropology, University of Vienna, Vienna, Austria; 2 Research Centre of Religion and Transformation in Contemporary Society, University of Vienna, Vienna, Austria; 3 QIMR Berghofer Medical Research Institute, Brisbane, Australia; 4 Faculty of Health, School of Biomedical Sciences, Queensland University of Technology (QUT), Brisbane, Australia

## Abstract

It is long known that inbreeding increases the detrimental effects of recessive sequence variants in “Runs of Homozygosity” (ROHs). However, although the phenotypic association of ROH has been investigated for a variety of traits, the statistical power of the results often remains limited as a sufficiently high number of cases are available for only a restricted number of traits. In the present study, we aim to analyze the association of runs of homozygosity with the trait “in-group ethnic favoritism”. This analysis assumes that if ethnic identity is important for an individual, that individual may tend to marry more frequently within their own group and therefore ROH are expected to increase. We hypothesize that an attitude preferring one’s own ethnic group may be associated with a stronger tendency of inbreeding and, as a result, with more and longer ROHs. Accordingly, we investigated the association between the attitude to someone’s own ethnicity and ROH, using the Wisconsin Longitudinal data (WLS, total N ~ 9000) as discovery data set and the Brisbane Twin data as replication data set (N ~ 8000). We find that both the number as well as the total length of homozygous segments are significantly positively associated with “in-group ethnic favoritism”, independent of the method used for ROH calculation.

**Supplementary Information:**

The online version contains supplementary material available at 10.1007/s10519-021-10053-z.

## Introduction

As DNA sequencing techniques have advanced, the interest in homozygosity, including the population history leading to autozygosity and its associations with other traits, has increased (Ceballos et al. 2018). The degree of homozygosity is measured in terms of “*runs of homozygous (ROH) DNA sequences*”, which are uninterrupted, identical DNA-sequences that emerge whenever two identical haploid copies of a sequence are inherited by both parents and, thus, brought together in one individual. The major mechanism leading to large homozygous segments in the genome is inbreeding, i.e. marriage among kin. Although the frequency of consanguineous marriages has become low in Western industrialized societies, in other parts of the world, the proportion of marriages among second or lower order cousins is still rather high (http://consang.net/index.php/Global_prevalence; Bittles and Neel 1994).

On the basis of ROHs, the history of a population can be reconstructed to a certain extent. To some degree, everyone inherits identical chromosomal segments from both parents. Homozygosity therefore provides a window to the individual and population demographic past (Ceballos et al. 2018). Smaller populations, for instance, have more ROHs than larger ones, admixed populations have fewer ROHs compared to the populations that have not been mixed, and inbred populations have longer ROHs compared to more outbred ones. The highest number and longest ROHs can be found in inbred populations that also have faced a bottle neck event (Ceballos et al. 2018).

It is well known that generally, inbreeding increases the detrimental effects of recessive sequence variants in ROHs (studies reviewed in Ceballos et al. 2018). However, although the phenotypic association of ROH has been investigated for a variety of traits (reviewed in Ceballos et al. 2018), the statistical power of the results often remains limited as sufficiently high number of cases are only available for a restricted number of traits. A study on 354,224 individuals from 102 cohorts reported a significant negative association between total length of ROH (equivalent to first order cousins) and four complex traits: body height, education, cognitive ability, and forced expiratory lung volume (Joshi et al. 2015). Abdellaoui et al. (2015) also reported that in higher educated individuals, the proportion of the genome consisting of runs of homozygosity is lower, which was mediated by the geographical distance between parental birthplaces. Recently, it was shown, using genetic data of 1.4 million individuals, that ROH are associated with deleterious changes in 32 out of 100 investigated traits (Clark et al. 2019). Particularly interesting are the striking effects on fertility—with ROH equivalent to the offspring of first cousins associated with a 55% decrease in the odds of having at least one child (Clark et al. 2019). The association of ROH with such diverse traits points to potentially complex associations of homozygosity.

We speculate that an attitude of ethnic “in-group favoritism” may lead to more in-group marriages and thus to an increase in genome– wide ROH. Accordingly, we investigated whether traits characterizing in-group ethnic favoritism, such as “*the importance of your ethnic group/nationality identity* “, favoring “A*partheid*”, “*white superiority*” and “*patriotism*” as well as rejecting “*multiculturalism*” and “Asian *immigration*” are associated with an increase of genome-wide ROH. As generally, in-group favoritism has an inherited component (varying greatly from 18 to 79% depending on the actual trait surveyed; Loehlin 1993; Lewis et al. 2014; Kandler et al. 2015), we assume that the partly inherited tendency of in-group favoritism should be detectable by an increase of ROH. ROH may thus indicate a history of in-group favoritism and in-group marriage.

We used the Wisconsin Longitudinal study (WLS) as discovery data set and the Brisbane Twin Study as replication data set.

## Methods

### Discovery Data Set: “Wisconsin Longitudinal Study”

The Wisconsin Longitudinal Study (WLS) is a long-term study of a random sample of men and women who graduated from Wisconsin high schools in 1957 and their siblings. The WLS panel started out with 10,317 members from the class of 1957. A second sample of 8734 randomly selected siblings of the original graduate panel were recruited for the study. Of these combined samples, 9027 individuals contributed saliva for genetic analysis (4556 individuals are related, as they are members of a family). In total 713,014 SNPs had been genotyped. (The Wisconsin Longitudinal Study genetic data is sponsored by the National Institute on Aging (Grant Numbers R01AG009775, R01AG033285 and R01AG041868) and was conducted by the University of Wisconsin). Detailed information on individual recruitment, genotyping, and quality control can be found at https://www.ssc.wisc.edu/wlsresearch/documentation/GWAS/Herd_QC_report.pdf. Data use agreement from the 30. May 2019; project: “Genome Wide Association Studies and Runs of Homozygosity”.

### Runs of Homozygosity

We calculated the runs of homozygosity (ROH) of the WLS genotypic sample using two different methods of ROH estimation:

(i) Runs of homozygosity according to Howrigan et al. (2011)

We calculated ROH on the basis of the recommendations of Howrigan et al. (2011) by first removing all SNPs with a minimal allele frequency of lower than 5% (MAF 0.05) and performing a “moderate SNP pruning” using a 50 SNP “window”, a 5 SNPs shift, and a VIF of 2. According to Howrigan et al. (2011), moderate SNP pruning and the threshold of 0.05 MAF leads to optimized results in the later calculation of ROH. MAF threshold of 0.05 and pruning resulted in a total of 133,442 SNPs, which we used for the calculations of ROH in a sliding window with a threshold of 50. We performed MAF removal, pruning and ROH calculation in PLINK (http://zzz.bwh.harvard.edu/plink/). (light pruning: plink—bfile WLS –indep 50 5 2—out WLS_Pruned; plink—bfile WLS_Pruned_F—homozyg-window-het 0—homozyg-snp 50—out WLS_Pruned_F_ROH).

(ii) Runs of homozygosity according to Clark et al. (2019)

Runs of homozygosity (ROH) were identified on the basis of SNPs with minor allele frequencies higher than 5%. In line with Clark et al. (2019), we used PLINK 1.9 with the following parameters to calculate ROH: homozyg-window-snp 50; homozyg-snp 50; homozyg-kb 1500; homozyg-gap 1000; homozyg-density 50; homozyg-window-missing 5; homozyg-window-het 1. In contrast to the method of Howrigan et al. (2011), no linkage disequilibrium pruning was performed (a detailed description can be found in Clark et al. (2019)).

To estimate effects due to the size of the ROH, we additionally estimated ROH according to Clark et al. (2019) with a “SNP-window” of 100 kb, 500 kb, 1000 kb, 2000 kb, 2500 kb, 3000 kb and 5000 kb (–homozyg-kb).

Both methods of ROH-calculation produced two measures of ROH each: (i) the total number of homozygotes segments (NSEG), and (ii) the sum of length of homozygotes segments (KB). In the supplement, we show the correlation of NSEG calculated according to Howrigan et al. (2011) vs. Clark et al. (2019) (Figure S1a) as well as the correlation of KB (Figure s1b).

#### Phenotypic Variable

We analyzed the association of (i) NSEG, and (ii) KB, respectively, and the phenotypic variable “importance of ethnicity” in terms of the answer to the question “*How important is your ethnic group/nationality identity*” surveyed in the years 2005–2006 and encoded on a 7 item scale from not important (1) to very important (7) (1: 12,038, 2: 1318, 3: 1044, 4: 1459, 5: 817, 6: 600, 7: 443).

Additionally, we included the following variables in our analysis: year of birth, sex (encoded as 1 = male; 2 = female), and highest education surveyed in the years 2005–2006 and 2011 (encoded as: 1 = no further education after high school mentioned; 2 = associate’s degree; 3 = bachelor's degree; 4 = master's degree; 5 = doctorate or professional degree).

### Analyses

We regressed the ordinal phenotype “importance of ethnicity” using the following four separate mixed ordinal models (R-library ordinal function clmm): “importance of ethnicity” regressing on sex, birth year, highest education and the first 10 principal components of the population structure (Abdellaoui et al. 2013), as well as (i) NSEG calculated according to Howrigan et al. (2011), (ii) NSEG calculated on basis of Clark et al. (2019), (iii) KB calculated according to Howrigan et al. (2011), or (iv) KB calculated according to Clark et al. (2019). To control for relatedness among individuals, family ID was included as a random factor in the models.

In addition, to avoid confounding effects of kinship and ancestry, we calculated the same models (but without the random factor) only including non-related as well as individuals of only European ancestry in our analyses (results presented in the supplement). Kinship and European ancestry were determined on the basis of genome-wide SNP data: Individuals closer related than third order cousins have been removed by KING (http://people.virginia.edu/~wc9c/KING/). Furthermore, to confine effects of ancestry, we only included individuals with > 99% European ancestry determined using ADMIXTURE (Alexander et al. 2009) with a K factor of 3 (Caucasians, Afro Americans and Others). This sample finally consists of 2740 male and 2884 female who are no closer related than third order cousin Europeans.

### Genome-Wide Regression

The association between NSEG and KB, respectively, and in-group ethnic favoritism was assessed by estimating how much of the variance of the phenotype *“importance of own ethnicity”* was accounted for by either NSEG or KB scores in each cohort as described in Mitchell et al. (2020). This was done using a logistic mixed model regression with either NSEG or KB as a predictor variable, while accounting for sex, year of birth, the first 10 genetic principal components (to account for residual population stratification), as well as for imputation run, a variable used to capture in-house cohort differences arising from differences in genotyping array and imputation, as fixed effects; relatedness among individuals was accounted for as a random effect with a genetic relatedness matrix. This analysis was implemented in GCTA 1.91.7 (Yang et al. 2011; Yang et al. 2014). Nagelkerke’s R^2^ was used to estimate the variance explained by the predictors. Significance values were calculated using a two-tailed Student’s t test. To correct for multiple testing error, the p-value threshold was adjusted by the number of independent tests (n = 4) before undergoing Bonferroni correction (α = 0.0125).

### Replication Data Set: “Brisbane Twin Study” (BTS)

We used the data from Australian twins, one sample of twins born before 1964 and surveyed in 1980, aged between 19 and 87 years (Martin et al. 1986), and a second sample of twin participants born 1965–1971 surveyed between 1989 and 1991 (Posner et al. 1996). The questions on the "in-group ethnic favoritism" used in our study are part of a survey on the general attitudes towards liberalism-conservatism and had been assessed in a Wilson and Patterson (1968) format in all surveys: The survey inventory was presented to participants as a short stimulus word or phrase and they were asked to respond positively, negatively, or neutrally to each. For all analyses, we only used the definite negative or positive answers but not the neutral answers.

As no explicit question on the importance of ethnic identity was included in the BTS, we used the 5 most related questions as phenotypes, each encoded as 0 = No and 1 = Yes for advocating the phenotype:

i) Apartheid (6714 No, 465 Yes)
ii) Multiculturalism (1105 = No, 5813 = Yes),
iii) White Superiority (7487 = No, 475 = Yes)
iv) Patriotism (833 = No, 6123 = Yes)
v) Asian immigration (2890 = No, 3421 = Yes).

The numbers of cases are drawn from the full data set, actual numbers for each model may vary according to the joint availability of the confounding variables. For further analysis, we recoded the variables “Multiculturalism” and “Asian immigration”, so that for all variables the more right-wing attitude was encoded as 1 and the more left-wing attitude as 0.

Genome-wide genotyping was previously performed using a range of genotyping arrays with standard imputation and quality control procedures as previously described (Medland et al. 2009). Only SNPs with a minor allele frequency ≥ 0.05 from chromosomes 1–22, X were included. Individuals with a genotypic missing rate ≥ 3%were excluded (plink -code: plink_1.90—bfile filexxx—chr 1–22, X—maf 0.05—mind 0.03—make-bed). Runs of homozygosity were calculated using the following plink 1.9 code (detailed description in Clark et al. 2019): plink_1.90 –bfile (basefilename_from_previous_step)—het—homozyg—homozyg-density 50—homozyg-gap 1000—homozyg-kb 1500—homozyg-snp 50—homozyg-window-het 1—homozyg-window-missing 5—homozyg-window-snp 50—ibc.

We included the following individuals in our analysis: in total 1464 female MZ twins, 607 male MZ twins, 920 female DZ twins, 464 male DZ twins, 1010 opposite sex DZ twins and 1722 other family members. Zygosity and family membership was encoded in one variable “*zygosity & kin*”: 1 = female MZ twins, 2 = male MZ twins, 3 = female DZ twins, 4 = male DZ twins, 5, 6 = opposite sex DZ twins, and 7 = non-twin kin, member of a family.

We calculated separate models for each of the five phenotypes regressing either on NSEG or KB, while controlling for age at the time of the survey, sex (encoded as 1 = male, 2 = female), years of education, year of survey, the 10 PCs, and kinship with family ID and *zygosity & kin* as random factors (respectively only family ID as random factor in the supplement). We performed the linear mixed models using the R libraries MASS and MuMIn (functions glmmPQL, std.coef). As the results from the Brisbane data are a replication of the findings from Wisconsin Longitudinal, we refrained from correction for multiple testing.

The association between the NSEG and KB scores and the 5 phenotypes characterizing in-group ethnic favoritism (listed above) was assessed by estimating how much of the variance in each phenotype was accounted by the either NSEG or KB scores (described above in “*Genome-wide regression* “and in Mitchell et al. 2020).

Furthermore, we calculated a Cholesky decomposition twin model, using only the twins, for each of the 5 binary phenotypes separately, including i) NSEG, and ii) KB, respectively, as covariates to estimate the additive genetic heritability using the R umx library. We carried out all analyses in R.3.6.3, PLINK 1.9 (Shaun Purcell, http://pngu.mgh.harvard.edu/purcell/plink/, GCTA 1.92.4beta2, KING 2.1.4 (Manichaikul et al. 2010) and admixture 1.3 (Alexander et al. 2009).

## Results

### Discovery Data Set: “Wisconsin Longitudinal Study”

**Fig. 1:**
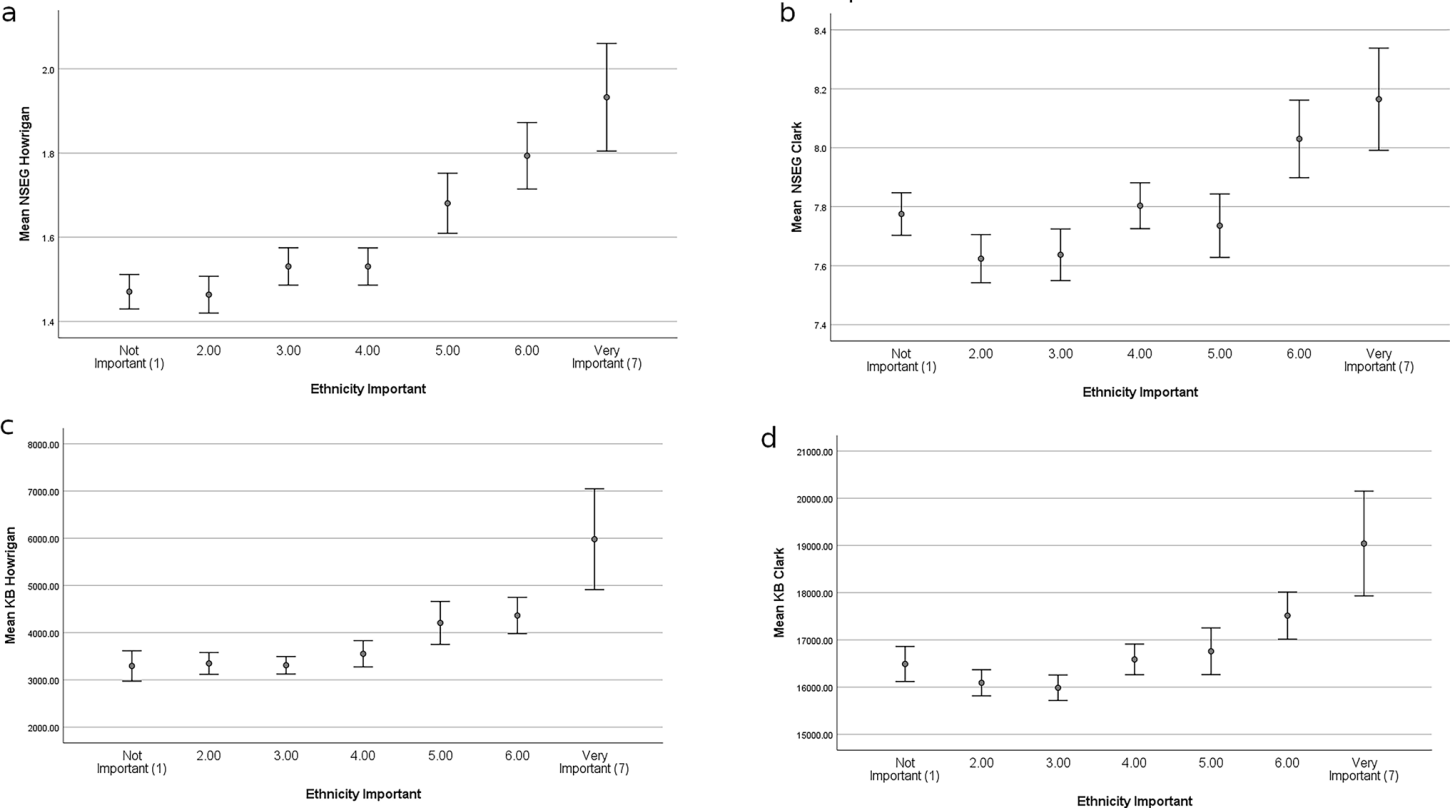
Importance of own ethnicity (varying from not important to very important) and NSEG (mean ± SE) (**a**) calculated on the basis of Howrigan et al. (2011) and (**b**) calculated on the basis of Clark et al. (2019). Importance of own ethnicity and KB (mean ± SE) calculated on the basis of Howrigan et al. (2011) (**c**) and Clark et al. (2019) (**d)**

We find that the importance of own ethnicity increases with increasing NSEG and KB, calculated both on the basis of Howrigan et al. (2011) (Fig. 1a, c) as well as Clark et al. (2019) (Fig. 1b, d), although NSEG and KB calculated on the basis of Clark et al. (2019) shows to some extent a curve–linear pattern (Fig. 1b, d). Also, by applying the ordinal mixed models, both, NSEG and KB, calculated on basis of Howrigan et al. (2011) as well as Clark et al. (2019), are significantly positively associated with increasing importance of own ethnicity (Table 1). However, the estimates and significances calculated on the basis of Howrigan et al. (2011) are higher than those calculated on the basis of Clark et al. (2019) (Table 1). Year of birth, being female and highest education are significantly negatively associated with the importance of own ethnicity, indicating that own ethnicity is less important for younger individuals, women and higher educated individuals (Table 1). Results remain virtually unchanged if non-kin and individuals of non-European ancestry are excluded from the analysis (supplement Table S1).

**Table 1:** WLS: Ordinal mixed models regressing the “*importance of own ethnicity*” on year of birth, sex, highest education and (i) NSEG, as well as ii) KB, respectively, calculated either on the basis of Howrigan et al. (2011) and Clark et al. (2019)

	ROH Howrigan			ROH Clark		
	Estimate	SE	ODDS	Estimate	SE	ODDS
NESG model						
Year birth	−0.034***	0.005	0.9670	−0.034	0.005	0.9664
Sex female (ref. male)	−0.121**	0.042	0.8864	−0.152***	0.044	0.8586
Highest education	−0.061***	0.018	0.9406	−0.06***	0.018	0.9416
**NSEG**	0.044***	0.018	1.0453	0.018*	0.007	1.0184
KB model						
Year birth	−0.034***	0.005	0.9668	−0.034***	0.005	0.9665
Sex female (ref. male)	−0.121**	0.042	0.8862	−0.137**	0.043	0.8717
Highest education	−0.06***	0.018	0.9418	−0.06***	0.018	0.9417
**KB**	0.006**	0.002	1.0006	0.005*	0.002	1.0005

Estimates of the ordinal mixed mode (*indicate significance level: *P < 0.05, **P < 0.01, ***P < 0.001); standard errors and Odds ratios

Additionally, in the regression analysis using GTCA, both NSEG and KB calculated on the basis of Howrigan et al. (2011) as well as NSEG calculated on basis of Clark et al. (2019), are significantly positively associated with the importance of own ethnicity after Bonferroni correction (Table 2). Again, effect sizes, variance explained, and significances are higher when NSEG and KB are calculated on the basis of Howrigan et al. (2011). However, NSEG and KB only explain a small proportion of the overall variance of the importance of ethnicity (Table 2). We found a heritability estimate of 15.1% for the importance of ethnicity.

**Table 2:** WLS: Association results of NSEG and KB, respectively, calculated on the basis of Howrigan et al. (2011) as well as Clark et al. (2019), on the importance of ethnicity

Predictor	Beta	Beta (SE)	% Variance expl	P
NSEG (Howrigan)	0.0405	0.0120	0.1667	0.0008*
KB (Howrigan)	5.0000E−06	0.0000	0.1231	0.0124*
NSEG (Clark)	0.0180	0.0071	0.0329	0.0117*
KB (Clark)	4.0000E−06	2.0000E−06	0.0788	0.0455

*Still significant after Bonferroni correction (P < 0.0125)

The analyses according to the length of homozygous segments (100 kb-5000 kb) in separate models revealed that although NSEG and KB (according to Clark et al. 2019) are significant in most of these models, after Bonferroni correction, only NSEG with a length of 2500 kb remains significant (supplement Table S2).

### Replication Data Set: Brisbane Twin Study

In the Brisbane twin sample, we find that higher ROH in terms of NSEG is significantly positively associated with being more patriotic and favoring Apartheid but significantly negatively associated with White superiority and Asian immigration, indicating lower favoritism of White superiority and higher acceptance of Asian immigration (Table 3). The same associations, albeit in part only marginally significant, with patriotism, favoring Apartheid and Asian immigration hold true for ROH calculated in terms of KB. KB but not NSEG is also significantly negatively associated with multiculturalism, thus indicating higher acceptance of multiculturalism. KB is not significantly associated with White superiority (Table 3). Models are similar whether family ID and zygosity (Table 3) or only family ID (supplement Table S3) is used as random factor.

**Table 3:** BTS: Regressing attitude (encoded as 1 = more right wing, 0 = more left wing) towards patriotism, white superiority, multicultural societies, Apartheid, and Asian immigration, separately, on age, sex, years of education, survey years as well as (i) NSEG and (ii) KB, with the random factors family ID and zygosity & kin on basis of a binomial error structure

	Patriotism			White sup			Multicultural			Apartheid			Asian im		
	Beta	P	SE	Beta	P	SE	Beta	P	SE	Beta	P	SE	Beta	P	SE
NSEG models															
Age	0.983	***	0.187	1.563	***	0.273	− 0.190		0.153	− 0.061		0.237	− 0.468	***	0.093
Sex female (ref. male)	0.623	***	0.155	− 3.161	***	0.232	− 1.585	***	0.138	0.762	***	0.224	− 0.147	•	0.087
Years education	− 0.378	*	0.161	− 2.163	***	0.261	− 1.639	***	0.158	− 2.405	***	0.242	− 1.996	***	0.100
Survey year	− 0.650	**	0.207	1.209	***	0.319	− 0.365	*	0.180	2.553	***	0.342	0.400	***	0.098
NSEG	0.506	**	0.170	− 0.730	**	0.269	− 0.139		0.156	1.198	***	0.238	− 0.200	*	0.091
KB models															
Age	0.988	***	0.187	1.519	***	0.272	− 0.187		0.153	− 0.124		0.238	− 0.463	***	0.093
Sex female (ref. male)	0.663	***	0.154	− 3.289	***	0.229	− 1.583	***	0.135	0.837	***	0.221	− 0.171	*	0.086
Years education	− 0.372	*	0.161	− 2.168	***	0.260	− 1.641	***	0.158	− 2.329	***	0.241	− 2.000	***	0.100
Survey year	− 0.648	**	0.207	1.247	***	0.318	− 0.365	*	0.181	2.497	***	0.339	0.402	***	0.098
KB	0.418	•	0.221	0.191		0.185	− 0.327	*	0.159	1.212	***	0.212	− 0.155	•	0.091

Beta values, standard errors significances indicated by level of significance: ^•^P < 0.1 (marginally significant), *P < 0.05, **P < 0.01, ***P < 0.001. Estimates for the 10 PCA are not shown

All phenotypes show a significant association with sex although the direction differs: women are more patriotic and favoring Apartheid more than men but otherwise are also more accepting towards multiculturalism and Asian immigration and less favoring White superiority. Signs and significances do not differ substantially in the KB and NSEG model as well as using different random variables (cf. Table 3 and supplement Table S3). Notably, in all models (Table 3, Table S3), education is significantly negatively associated with each phenotype, indicating a generally more left-wing attitude among the higher educated. A more recent survey year is associated with lower patriotism and higher acceptance of multiculturalism but also with more favoring White superiority, Apartheid and less tolerance towards Asian immigration, both in the KB and the NSEG model (Table 3). The same holds true in the models with different random factors (supplement Table S3) except attitude towards White superiority.

In the regression analysis using GCTA, NSEG and KB are significantly positively associated with approval of White superiority (NSEG, P = 0.025, KB, P = 0.012). Approval of Apartheid is marginally significantly positively associated with NSEG (P = 0.054) but negatively associated with KB (P = 0.014). The other 3 phenotypes show no significant association with any measure of ROH (Table 4). The proportion of variance explained by NSEG and KB, however, is very low (< 1%). Heritability has been estimated between 24.4% and 41.6% (Table 5). The inclusion of NSEG or KB did not change the heritability estimates substantially (data not shown).

**Table 4:** Regression analysis using GCTA with NSEG and KB as covariates, calculated separately on the 5 phenotypes (i.e. patriotism, as well as attitude towards white superiority, multiculturalism, Apartheid and Asian immigration)

Predictor	Phenotype	Beta	Beta (SE)	% Variance expl	P
KB	Apartheid	9.10E − 05	3.70E − 05	0.14200	0.014
KB	Multicultralism	4.40E − 05	5.30E − 05	0.01580	0.407
KB	White superiority	5.00E − 05	3.20E − 05	0.14780	0.012
KB	Patriotism	1.80E − 05	5.10E − 05	0.00314	0.724
KB	Asian immigration	1.00E − 04	6.90E − 05	0.04280	0.147
NSEG	Apartheid	− 1.23E − 03	1.36E − 04	0.19520	0.054
NSEG	Multicultralism	− 1.22E − 03	1.19E − 03	0.02130	0.306
NSEG	White superiority	6.00E − 03	2.72E − 03	0.11925	0.025
NSEG	Patriotism	1.06E − 03	1.96E − 03	0.00691	0.589
NSEG	Asian immigration	1.28E − 03	1.87E − 03	0.01198	0.492

**Table 5:** ACE models (squared standardized estimates) of the 5 binary phenotypes

	A	C	E
ACE Patriotism	0.416	0.000	0.584
ACE White superiority	0.244	0.000	0.755
ACE Multiculturalism	0.253	0.114	0.634
ACE Apartheid	0.298	0.000	0.702
ACE Asian migration	0.335	0.172	0.493

## Discussion

In the WLS, we find a significant positive association between ROH and in-group ethnic favoritism irrespective of the method used to calculate ROH, or whether the number of homozygous segments (NSEG) or the length of homozygous segments (KB) are used as the indicator for homozygosity. However, estimates calculated on the basis of Clark et al. (2019) are lower and less significant compared to estimates calculated on the basis of Howrigan et al. (2011). This difference between methods might be due to the fact that Clark et al. (2019) use a fixed “SNP window” for the estimation of ROH, whereas Howrigan et al. (2011) use pruned LD data, which may lead to more clearly separated regions of ROH and therefore to a more pronounced signal.

Because Clark et al. (2019) use a fixed “SNP window” for the estimation of ROH, we were able to investigate the effects of different window sizes on the association of NSEG, KB with the phenotypes. We found that changing the window leads to associations in the same direction, but with estimates of different size and significance (see supplement Table S2); estimates for NSEG increase with increasing size of SNP window whereas estimates for KB remain similar with increasing SNP window size. Although applying strict Bonferroni correction for the testing of multiple SNP-windows (supplement Table S2) leads to non-significant results, we assume that with a larger sample size, we would be able to detect significant results.

In the logistic regression model conducted in GCTA, both NSEG and KB (calculated according to Howrigan et al. (2011) as well as Clark et al. (2019)) are significantly positively associated with “importance of ethnicity”, albeit both NSEG and KB explain only a small proportion of the phenotypic variance. Overall, SNPs are estimated to account for 15.1% of the phenotypic variance. The principle results on the basis of the WLS study did not change if we included all individuals controlling for population structure or if we only included individuals of European ancestry and non-kin in our analysis (see supplement).

The results of the Brisbane twin studies are partly concordant with the results from the WLS data set. In the Brisbane twin study, in accordance with the positive association of ROH and in-group ethnic favoritism found in the WLS, more right wing positions towards patriotism, and Apartheid (i.e. approval of these attitudes) are significantly or marginally significantly positively associated with one measure of ROH (NSEG or KB) irrespective of whether family ID and zygosity & kin or only family ID are used as random factors. On the contrary, higher ROH is associated with a more left-wing attitude towards multiculturalism and Asian immigration. The results for the attitude towards white superiority are inconsistent as NSEG is significantly negatively but KB is not significantly associated with favouring white superiority.

A possible explanation for the partially differing results obtained from the WLS and the Brisbane Twin Study may be found in differences in survey questions and scales. There are seven scales in the WLS data set that allow for more moderate responses compared to a binary scale in the Brisbane data set that covers only extreme positions. These fewer increments correspond with lower “signal” (estimates and significance), which may lead to the observed differences. Furthermore, our sample size may be too small to detect any robust effects (Keller et al. 2012; Johnson et al 2016). In addition, the “homozygotes structure” of both data sets is substantially different, although the plot KB vs. NSEG indicates to some extent an admixed population (Ceballos et al. 2018). Compared to the Brisbane data set, the WLS data set includes more individuals who are extreme on either NSEG, KB or both, i.e. individuals who are from small populations (high NSEG), have a recent history of consanguinity (high KB), or both (see supplement Figure S2). As the Brisbane data set is “less extreme” in this regard, this may also explain the less significant association of ROH and the surveyed phenotypes.

In contrary to the WLS data, in the Brisbane data, the results from the regression analysis differ in sign and/or significance in part from the results obtained by GCTA (namely for *KB-Multiculturalism, KB-Asian Immigration, NSEG-White Superiority, NSEG-Asian Immigration*). These differences may be caused by the estimation of the genetic relatedness (GRM) matrix by GCTA, which additionally controls for degrees of genetic relatedness and thus more accurately accounts for relatedness than the regressions in R. By including the family ID as a random effect, all family members (this could mean parents, twins or siblings) are treated equally, whereas the GRM used in GCTA accounts for these specific relationships so that they are modelled differently depending on how much genetic information they share.

The association between in-group ethnic favoritism and ROH may be caused to some extent by an “underlying association” between ROH and Socio-economic status (SES), as a negative association between ROH, education and general cognitive ability has been already shown (Joshi et al. 2015; Abdellaoui et al. 2015). However, in the WLS, we only found a marginally significant negative association between income and NSEG and KB (supplement Table S4a, b), respectively, and no significant association of NSEG or KB and education (data not shown), indicating only a weak association between SES and ROH: individuals with high ROH tend to have lower levels of income. The lack of any significant association of ROH and education may be due to the fact that all Wisconsin participants are at least A-level educated leading to a lack of variability in educational attainment in the sample.

In the Brisbane data set, we also did not find a significant association between ROH and education, which is generally associated with a more left-wing attitude (data not shown), but we found interactions between education, ROH and the measures of in-group ethnic favoritism. Albeit the general results for white superiority are inconsistent, a positive attitude towards white superiority regressed significantly positive on the interaction of education and NSEG—indicating that with increasing education, higher ROH is associated with a more “right wing attitude” towards white superiority (supplement Table S5). However, multiculturalism is different as increasing education and ROH are associated with a more liberal attitude towards multiculturalism (supplement Table S5). The results remain consistent whether Family ID & kin or only family ID is included as random factor. Thus, overall, we cannot exclude SES effects and interactions between SES, ROH and in-group ethnic favoritism, but on the basis of our data, we are not able to make any final conclusions.

We did search for some additional “phenotypic evidence” that a more in-group ethnic favoritism attitude may foster inbreeding and thus lead to an increase in homozygosity. In support of our assumptions, on the basis of data from 18 countries from the World Value Survey (supplement Table S6), we found that a more restrictive attitude towards ethnic diversity and the percentage of first and second order cousin marriage is positively correlated (R = 0.55, P = 0.023; supplement Figure S3) indicating that indeed there might be an association between ethnocentrism and inbreeding.

We know from twin studies that in-group favoritism is partly genetic, albeit the contribution of genetics to in-group ethnic favoritism and in-group favoritism in general is less clear compared to other traits such as for instance political attitude (Hatemi and Dermot 2012). The variance explained in twin studies varies greatly from 18 to 79% depending on the actual trait surveyed and the precise definition of “in-group” (Loehlin 1993; Lewis & Bates 2010; Orey and Park 2012; Lewis et al. 2014; Kandler et al. 2015). Hence, the partly inherited attitude towards other ethnicities may influence the genomics of homozygosity and may thus provide some kind of “genomic feedback loop”. So in-group ethnic favoritism could be an example where a partially inherited trait may lead to “genomic change” in terms of an increase in homozygosity, providing evidence for a cultural-genetic co-evolution (Richerson et al. 2010).

Alternatively, although very unlikely and virtually undetectable with our sample size (Keller et al. 2011, 2012), ROH might directly influence in-group ethnic favoritism so that the association would exist primarily not because of a history of within-group mating, but because more homozygous individuals show more ethnocentrism due to any gene–phenotype connection.

Although sample size is still too small to detect consistent effects as it has happened, for instance, in the analysis of homozygosity and schizophrenia (Keller et al. 2012; Johnson et al 2016), we conclude that in-group ethnic favoritism is associated with higher ROH, a finding that to only some extent is confirmed by data from the Brisbane Twin Study.

## Supplementary Information

Below is the link to the electronic supplementary material.

Supplementary file1 (DOCX 179 KB)

## Data Availability

Data from Wisconsin Longitudinal is after IRB review and proposal submission available from “Wisconsin Longitudinal”.
