# Supplementary material for: Ethnic Identity and Genome Wide Runs of Homozygosity
Source: Behav Genet. 2021 Mar 16;51(4):405–13. doi: 10.1007/s10519-021-10053-z (PMC8225526; doi:10.1007/s10519-021-10053-z)
Supplement: Supplementary file 1 — Supplementary file1 (DOCX 179 KB) [file 10519_2021_10053_MOESM1_ESM.docx]

**Supplement:**

**
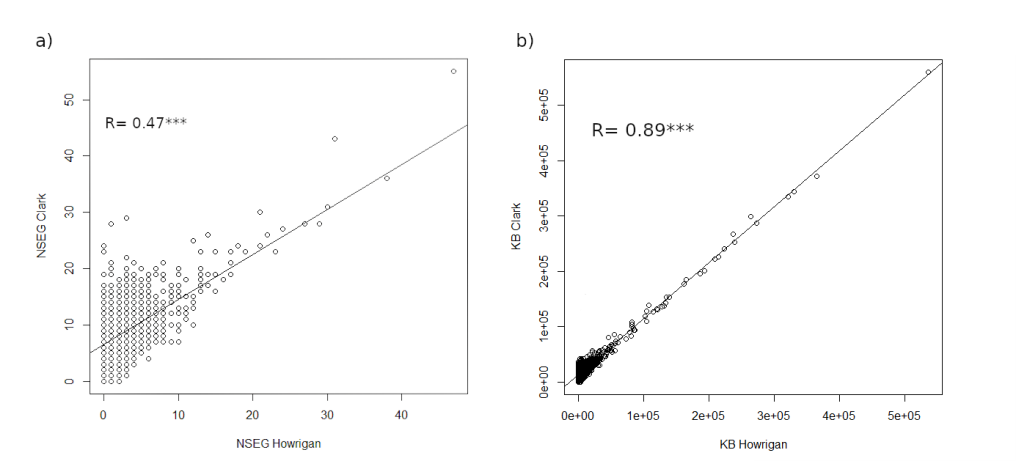
**

Figure S1 a) Correlation of NSEG calculated according to Howrigan vs. Clark ; b) Correlation of KB calculated according to Howrigan vs. Clark


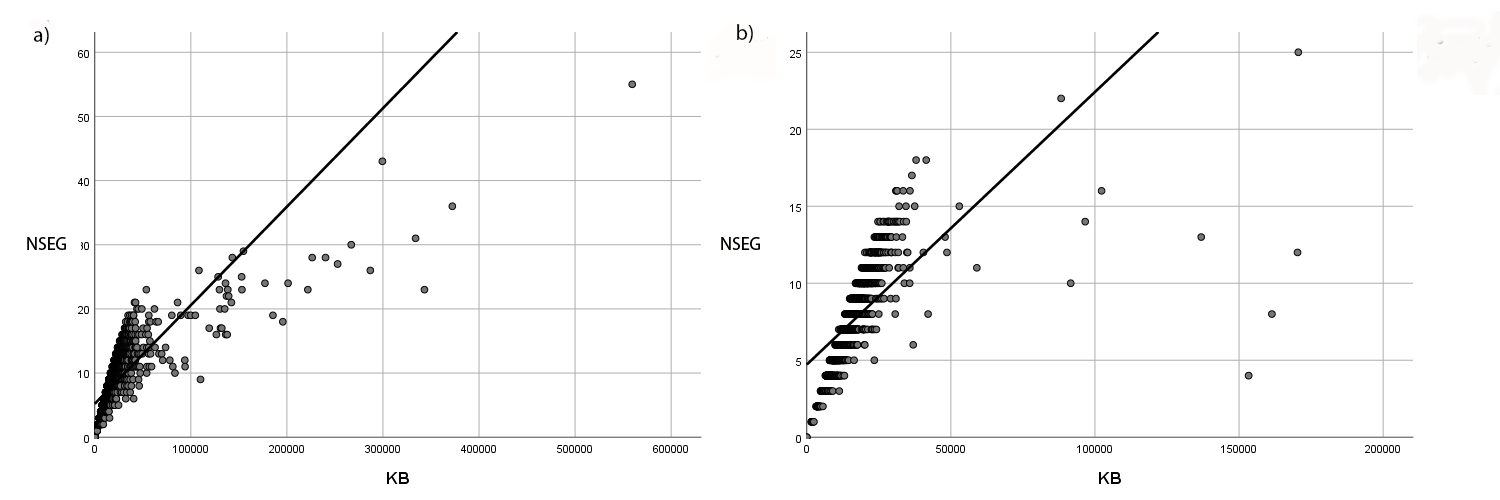


Figure S2) a) KB vs. NSEG for the Wisconsin sample and b) KB vs. NSEG for the Brisbane sample. Calculation of NSEG and KB same for both data sets; on basis of Clark et al. (2019).

Figure S3) Correlation between the prevalence of consanguinity and the attitude towards ethnic diversity, data from the world value survey.

Table S1) Parameter estimates of the ordinal mixed models regressing the “*importance of own ethnicity*” on year of birth, sex, highest education for individuals of European ancestry and no – kin only; for i) NSEG, as well as ii) KB, respectively, calculated either on the basis of Howrigan et al. (2011) and Clark et al. (2019). Estimates for the 10 PCA are not shown. * indicate significance level: * (P < 0.05), ** (P < 0.01), *** (P < 0.001).

|  |  | **Ethnicity ROH Howrigan** | **Ethnicity ROH Clark** |
| --- | --- | --- | --- |
| **NESG Model** | year birth | -0.0294*** | -0.0307*** |
|  | sex female (ref. male) | -0.1238. | -0.1822* |
|  | highest education | -0.0255 | -0.0212 |
|  | **NSEG** | 0.0788*** | 0.0352** |
| **KB Model** | year birth | -0.0298*** | -0.0302*** |
|  | sex female (ref. male) | -0.1254. | -0.1432 |
|  | highest education | -0.02 | -0.0194 |
|  | **KB** | 0.0088* | 0.0005 |

Table S2) Estimates and significances for NSEG and KB according to Clark et al. (2019) and different length of homozygoteous segments (not corrected multiple testing).

|  | **NSEG** | **KB** |
| --- | --- | --- |
| 100 KB | 0.12315 | 0.00034** |
| 500 KB | 0.28755. | 0.00031* |
| 1000 KB | 0.28755. | 0.00031* |
| 2000 KB | 2.29099* | 0.00041* |
| 2500 KB | 4.21455** | 0.00045* |
| 3000 KB | 4.18285* | 0.00041* |
| 5000 KB | 5.04047. | 0.00039. |

Table S3) BTS: Regressing attitude (encoded as 1 = more right wing, 0 = more left wing) towards patriotism, white superiority, multicultural societies, Apartheid, and Asian immigration, separately, on age, sex, years of education, survey years as well as i) NSEG and ii) KB, with the random factors family ID on basis of a binomial error structure. Beta values, standard errors significances indicated by level of significance: * (P < 0.05), ** (P < 0.01), *** (P < 0.001)). Estimates for the 10 PCA are not shown.

|  |  | **beta** | **P** | **Std. Error** | **beta** | **P** | **Std. Error** | **beta** | **P** | **Std. Error** | **beta** | **P** | **Std. Error** | **beta** | **P** | **Std. Error** |
| --- | --- | --- | --- | --- | --- | --- | --- | --- | --- | --- | --- | --- | --- | --- | --- | --- |
| **NSEG Models** | **age** | 0.654 | *** | 0.158 | 1.806 | *** | 0.213 | -0.097 |  | 0.128 | -0.939 | *** | 0.204 | -0.450 | *** | 0.083 |
|  | **sex female (ref. male)** | 0.429 | ** | 0.136 | -1.768 | *** | 0.201 | -1.357 | *** | 0.123 | 0.740 | *** | 0.196 | -0.143 | ** | 0.079 |
|  | **years education** | -0.361 | * | 0.141 | -2.116 | *** | 0.237 | -1.504 | *** | 0.145 | -2.813 | *** | 0.235 | -1.805 | *** | 0.092 |
|  | **survey year** | -0.124 |  | 0.156 | -0.663 | ** | 0.237 | -0.546 | *** | 0.142 | 0.682 | ** | 0.239 | 0.306 | *** | 0.084 |
|  | **NSEG** | 0.271 | . | 0.148 | -1.209 | *** | 0.237 | -0.164 |  | 0.139 | 0.381 | . | 0.209 | -0.171 | * | 0.082 |
| **KB Models** | **age** | 0.659 | *** | 0.159 | 1.700 | *** | 0.210 | -0.091 |  | 0.129 | -0.975 | *** | 0.204 | -0.448 | *** | 0.083 |
|  | **sex female (ref. male)** | 0.455 | *** | 0.135 | -1.997 | *** | 0.197 | -1.357 | *** | 0.121 | 0.695 | *** | 0.194 | -0.165 | * | 0.078 |
|  | **years education** | -0.357 | * | 0.141 | -2.119 | *** | 0.237 | -1.505 | *** | 0.145 | -2.819 | *** | 0.235 | -1.808 | *** | 0.092 |
|  | **survey year** | -0.120 |  | 0.156 | -0.645 | ** | 0.236 | -0.544 | *** | 0.142 | 0.653 | ** | 0.239 | 0.306 | *** | 0.085 |
|  | **KB** | 0.196 |  | 0.187 | 0.089 |  | 0.192 | -0.305 | * | 0.152 | 0.912 | *** | 0.202 | -0.117 |  | 0.082 |

Table S4a) Yearly income regressing on sex, birth year and NSEG calculated on basis of Howrigan et al.

|  | **Value** | **Std.Error** | **t-value** | **p-value** |
| --- | --- | --- | --- | --- |
| (Intercept) | 25914.5 | 10855.71 | 2.387179 | 0.017 |
| sex female (ref. Male) | -38870.5 | 2288.26 | -16.98689 | 0 |
| Birth_Year_z_brdxdy | 1069.9 | 269.29 | 3.973037 | 0.0001 |
| NSEG_HOWR | -1196.3 | 660.32 | -1.811705 | 0.0702 |
| DF = 1974 |  |  |  |  |
| StdDev: | 30474.12 |  |  |  |

Table S4b) Yearly income regressing on sex, birth year and KB calculated on basis of Howrigan et al.

|  |  |  |  |  |
| --- | --- | --- | --- | --- |
|  | **Value** | **Std.Error** | **t-value** | **p-value** |
| (Intercept) | 24585.4 | 10790.8 | 2.278363 | 0.0227 |
| as.factor(Sex_ResPondent_z_sexrsp)2 | -38858 | 2288.3 | -16.981153 | 0 |
| Birth_Year_z_brdxdy | 1073.2 | 269.25 | 3.985886 | 0.0001 |
| KB_HOWR | -0.2 | 0.1 | -1.877347 | 0.0606 |
| DF = 1974 |  |  |  |  |
| StdDev: | 30432.21 |  |  |  |

Table S5) Patriotism, White Superiority, Multiculturalism, Apartheid and Asian Immigration regressing on NSEG, education and the interaction of NSEG and Education, controlling for sex and birth year and the 10PCA (not shown). Beta values and significances.

|  | **NSEG** | **P** | **Education** | **P** | **NSEG x Educ** | **P** |
| --- | --- | --- | --- | --- | --- | --- |
| **Patroitism** | 0.593 |  | -0.335 |  | -0.096 |  |
| **White Superiority** | **-9.185** | ******* | -6.29081 | *** | **9.74956** | ******* |
| **Multiculturalsim** | **2.832** | ******* | -0.210838 |  | **-3.476718** | ******* |
| **Apartheid** | 1.003 |  | -2.508182 | *** | 0.233349 |  |
| **Asian Immigration** | 0.365 |  | -1.714835 | *** | -0.649118 |  |

Table S6) Prevalence of consanguinity and the attitude to ethnic diversity. Data from the world value survey. A higher mean value on the attitude towards ethnic diversity means a more restrictive attitude.

| **country** | **% consanguinity** | **attitude ethnic diversity** |
| --- | --- | --- |
| Jordan | 41.64 | 8.1112 |
| Egypt | 32.8 | 5.9935 |
| Hungary | 0.48 | 5.1469 |
| Morocco | 19.9 | 5.0745 |
| Spain | 4.1 | 5.0570 |
| Italy | 1.1 | 4.6457 |
| Australia | 0.85 | 4.5547 |
| Norway | 0.7 | 4.5178 |
| U.S.A. | 0.2 | 4.2042 |
| Chile | 0.7 | 3.8813 |
| Argentina | 0.2 | 3.7540 |
| Malaysia | 7.6 | 3.7350 |
| South Africa | 0.7 | 3.5127 |
| Brazil | 1.3 | 3.5005 |
| Mexico | 0.3 | 3.4237 |
| Sweden | 0.8 | 3.3863 |
| Indonesia | 17.8 | 2.7092 |
